# Blood arsenic levels and the risk of familial breast cancer in Poland

**DOI:** 10.1002/ijc.32595

**Published:** 2019-08-26

**Authors:** Wojciech Marciniak, Róża Derkacz, Magdalena Muszyńska, Piotr Baszuk, Jacek Gronwald, Tomasz Huzarski, Cezary Cybulski, Anna Jakubowska, Michał Falco, Tadeusz Dębniak, Marcin Lener, Oleg Oszurek, Katherine Pullella, Joanne Kotsopoulos, Ping Sun, Steven A. Narod, Jan Lubiński

**Affiliations:** ^1^ Department of Genetics and Pathology Pomeranian Medical University Szczecin Poland; ^2^ Read‐Gene SA Grzepnica Poland; ^3^ Department of Clinical Genetics and Pathology University of Zielona Góra Zielona Góra Poland; ^4^ Radiation Oncology Department West Pomeranian Oncology Center Szczecin Poland; ^5^ Women's College Research Institute Toronto Ontario Canada; ^6^ Dalla Lana School of Public Health University of Toronto Toronto Ontario Canada

**Keywords:** blood, cancer, cohort study, familial breast cancer, prospective study, Poland, arsenic, cancer risk

## Abstract

Arsenic is recognized as a potent carcinogen at high concentrations, but the relationship between environmental arsenic and breast cancer risk has not well been studied. Most research has focused on the effect of arsenic in populations with high endemic exposure, and not in populations with arsenic levels within normal limits. We sought to determine if blood arsenic levels predict the risk of breast and other cancers risk among women in northern Poland. The cohort consisted of 1,702 healthy women, aged 40 and above, identified between 2010 and 2017. Blood arsenic level was determined by inductively coupled plasma mass spectrometry. After an average of 4.5 years of follow‐up (range 0.7–7.3 years), there were 110 incident cases of cancer diagnosed in the cohort, including 68 cases of breast cancer. Women in the highest quartile of arsenic had a highly significant 13‐fold increased risk of developing breast cancer, compared to women in the lowest quartile (hazard ratio [HR] = 13.2; 95% confidence interval [CI] 4.02–43.0). Results were similar for arsenic and all incident cancers (HR quartile 4 *vs*. quartile 1 = 13.3; 95% CI 4.78–37.0). If confirmed, our study suggests that the blood arsenic level may be a useful predictive marker of cancer risk in women.

## Abstract 2

**What's new?**

Arsenic has long been recognized as a potent carcinogen at high concentrations. But can it affect cancer risk at “normal,” environmental concentrations? In this Polish study, the authors found that women whose blood levels of arsenic were in the highest quartile had a 13‐fold increased risk of developing breast cancer, compared to women in the lowest quartile. If confirmed in further studies, these results suggests that blood arsenic level may be a useful predictive marker of cancer risk in women.

## Introduction

The lifetime risk of breast cancer among women in Poland is approximately 6% and efforts are underway to better individualize risk, that is, to identify factors that allow us to stratify women into various risk categories. At the genetic level, these include mutations in *BRCA1*, *PALB2*, *CHEK2*, *NBN* and other susceptibility genes as well as single nucleotide polymorphism profiles which can be used to generate personalized risk scores.^1^, ^2^ For many women with a family history of cancer, a mutation is not found, but they remain at increased level of risk based on their family history alone. For these women, the extent of the risk increase in the face of a negative genetic test has not been well explored.

Over the past 50 years, there has been considerable attention paid to various measures of diet and breast cancer risk in prospective studies.^3^ Two approaches to quantifying exposure include measuring dietary intake using food frequency questionnaires or measuring a biomarker or nutrient within a blood component (i.e., serum, plasma or whole blood).^4^ In Poland, we are constructing a biomarker resource bank, along with a companion clinical database, which will eventually contain information on 7,000 women who have received genetic counseling at our institution (Pomeranian Medical University) because of a family history of breast cancer. The database will include 2,000 women with a *BRCA1* mutation and 5,000 women who do not have a *BRCA1* mutation. Study participants are cancer‐free at inception and are followed prospectively to identify new cases of cancer. To date, we have enrolled 1,700 noncarrier women and we have followed these women for an average of 4.5 years.

In the current study, we use the biorepository to address the question of blood arsenic as a possible risk factor for cancer. Arsenic has long been recognized by International Agency for Research on Cancer as a *bona fide* carcinogen for cancers of the skin, bladder and lung, but most studies to date have been based on highly exposed populations and little attention has been paid to the possible carcinogenic effect of low levels of arsenic.^5^ There has been preliminary data which suggest that arsenic may have an impact on breast cancer risk, although the findings are inconclusive.^6^, ^7^ There is limited data surrounding the relationship between arsenic exposure and subsequent breast cancer risk. There are 17 publications that have evaluated the relationship between arsenic and breast cancer. Four ecological studies evaluated soil, water or rice contamination with arsenic and local breast cancer rates.^8^, ^9^, ^10^, ^11^ Eleven case–control studies quantified either toenail, hair, blood or urinary arsenic species in breast cancer patients and controls.^12^, ^13^, ^14^, ^15^, ^16^, ^17^, ^18^, ^19^, ^20^, ^21^ Generally, the results show a weak or negative correlation with exceptions of reports by Wadhwa *et al*., and Joo *et al*., who found arsenic levels to be significantly higher in hair of breast cancer patients as compared to unaffected controls.^21^, ^22^ There have been no prospective studies that have examined blood arsenic levels and breast cancer risk. However, there have been two prospective studies examining the impact of ambient arsenic on breast cancer risk, one prospective study comparing toenail arsenic concentrations pre and post breast cancer diagnosis and one study that used rice consumption as a proxy for arsenic exposure.^8^, ^18^, ^23^, ^24^ In the latter study, Zhang *et al*. evaluated the association between long‐term rice consumption (a potential source of inorganic arsenic) and cancer risk in the Nurses' Health Studies and Health Professional Follow‐up Study.^8^ They reported no association between long‐term rice intake and overall cancer incidence of breast cancer. We prospectively evaluated the relationship between total blood arsenic and breast cancer risk in this cohort of 1,700 women with a family history of the disease but no inherited *BRCA1* mutation.

## Materials and Methods

### Study subjects

The study subjects were women aged 40 and above who had received genetic counseling and genetic testing at the Pomeranian Medical University between September 2010 and April 2017. No woman had been diagnosed with breast cancer or another type of cancer at the time of study entry. All study subjects provided written informed consent to participate in the study and all agreed to provide a blood specimen for research purposes. The study protocol was approved by the research ethics board of the Pomeranian Medical University. At the first outpatient clinic visit, a blood sample was taken for genetic testing for three founder mutations in *BRCA1*. In addition, a separate aliquot of 10 mL of whole blood was taken for research purposes and stored at −80°C. Blood samples were taken between 8 a.m. and 2 p.m. from Monday to Friday. The patients were requested not to eat nor to drink water for 4 hrs prior to the venipuncture.

Subjects completed a detailed questionnaire which included information about family history of cancer, age, smoking, hormones usage, personal medical history including adnexectomy and breast cancer screening history.

All subjects were tested for the three *BRCA1* mutations (c.5266dupC‐5382insC; c.181T>G‐300T>G; c.4035delA‐4153delA). Women with a *BRCA1* mutation were excluded from our study and will be the subject of a separate report.

### Arsenic measurement

Total arsenic concentration in blood samples was measured by inductively coupled plasma mass spectrometer (ICP‐MS), using the Elan Dynamic Reaction Cell‐e (PerkinElmer, Waltham, MA) instrument. Arsenic was measured in dynamic reaction cell mode with oxygen (Messer, O2 purity >0.9999) as a reaction gas. Under these conditions, arsenic forms oxides within the cell (AsO+) which can be detected at m/z 91, known to be free from spectral interferences. To compensate for instrument drift and matrix effects, rhodium was set as internal standard. All the parameters of Elan Dynamic Reaction Cell‐e used during measurement are available upon request.

The blank reagent consisted of high purity water (>18 MΩ), tetramethylammonium hydroxide (AlfaAesar, Haverhill, MA), Triton X‐100 (PerkinElmer), n‐butanol (Merck, Kenilworth, NJ) and disodium EDTA (Sigma Aldrich, St. Louis, MO). Calibration curve standards (0.1 μg/L; 0.,2 μg/L; 0.5 μg/L; 1.0 μg/L; 2.0 μg/L) were prepared by diluting stock solution (50 μg/L) of 10 mg/L Multi‐element Calibration Standard 3 (PerkinElmer Pure Plus) with blank reagent. The matrix matched calibration method was used. The correlation coefficients for arsenic calibration curves were greater than 0.999.

The accuracy of the method was validated using three different certified reference materials: National Institute of Standards and Technology 955c (Gaithersburg, MD) and Plasmonorm Whole Blood Level 1 (Clincheck, Germany). First, the level of the National Institute of Standards and Technology 955c reference material has been taken into consideration by analytics. The reference concentration value for arsenic in SRM955c Caprine Blood, Level 1 reported by National Institute of Standards and Technology is 2.07 ± 0.67 μg/L. These results are far away from values reported by our analytical laboratory and other authors.^25^ Analysis conducted with neutron activation analysis^26^ clearly identified that concentration of arsenic is nearly seven‐fold lower compared to the National Institute of Standards and Technology (0.28 ± 0.06 μg/L) which closely agrees with our results.

### Statistical analysis

There were 1,702 subjects enrolled in the follow‐up study; subjects were cancer‐free at baseline and did not carry a founder *BRCA1* mutation. Information on incident cancers was retrieved from the medical records and review of the pathology records of the treating Pomeranian hospitals. Subjects were classified into quartiles according to the blood arsenic level determined at the single measurement ensuring an equal number of women in each quartile. Women were followed from the date of blood draw or age 40 (whichever came last) to the first of breast cancer, death from another cause, or January 1, 2018. The annual incidence rates were calculated by comparing the number of events to person‐years of observation. Standardized incidence ratios were constructed by comparing the calculated age‐specific breast cancer rates to the Polish national breast cancer rates. The cumulative incidence of breast cancer in the entire cohort and in each of the four quartiles was estimated to 5 years, based on the Kaplan‐Meier method. Crude differences in cumulative incidence by arsenic quartile were tested for statistical significance using the log‐rank test. A multivariate hazard ratio (HR) was generated using the multivariate Cox model for arsenic by quartile (using the lowest quartile as the reference), adjusting for age at blood draw (<50, ≥50), smoking (ever/never), number of first‐degree relatives with breast cancer (2 or more, 1, *vs*. 0), oophorectomy (yes/no) and hormone replacement therapy use (yes/no). In a secondary analysis, we repeated the analysis using all cancers as the outcome. In this secondary analysis, patients were followed from the date of blood draw until the first cancer, death from another cause or January 1, 2018.

## Results

The cohort consisted of 1,702 women. None of the women had a prior history of cancer, but 34% had a family history of breast cancer and 7% had a family history of ovarian cancer. Of the 1,702 women, 58 had two or more first‐degree relatives with breast cancer, 515 had a single first‐degree relative with breast cancer and 1,129 had no first‐degree relative with breast cancer. Nine women had two or more first‐degree relatives with ovarian cancer, 117 had a single first‐degree relative with ovarian cancer and 1,576 had no first‐degree relative with ovarian cancer. A single arsenic measurement was available for each woman and, on average, 54 months had elapsed between the date of the blood draw and the date of arsenic measurement (range 8–88 months). The mean arsenic levels for different subgroups are presented in Table 1. The mean age at blood draw was 55.2 years (range 35.4–83.2 years) and 35% were current or past smokers.

**Table 1:** Characteristics of 1,702 women in the cohort

Characteristic	*n* (%)	Mean arsenic level, μg/L (range)
Age		
<50	549 (32)	1.04 (0.10–48.4)
50–59	583 (34)	1.08 (0.04–13.4)
60+	570 (33)	1.26 (0.07–20.1)
Smoking status		
Current/past	589 (35)	1.12 (0.06–13.4)
Never	1,113 (65)	1.13 (0.04–48.4)
Number of first‐degree relatives with breast cancer		
0	1,129 (66)	1.12 (0.06–20.10)
1	515 (30)	1.15 (0.04–48.39)
≥2	58 (4)	0.97 (0.04–13.4)
Hormone replacement therapy use		
No	949 (56)	1.08 (0.04–48.39)
Yes	712 (42)	1.19 (0.08–20.10)
Missing	41 (2)	0.91 (0.08–1.98)
Oophorectomy		
No	1,618 (95)	1.13 (0.04–48.39)
Yes	73 (4)	1.05 (0.06–7.09)
Missing	11 (<1)	1.03 (0.45–2.70)

The women were followed for an average of 4.5 years from the date of the blood draw (range 0.7–7.3). Collectively, the 1,702 women contributed 7,731 person‐years of follow‐up. Over the entire follow‐up period, there were 110 incident cases of cancer diagnosed in the cohort, including 68 cases of breast cancer, 6 cases of ovarian cancer and 36 cases at other sites (Table 2). Based on the 68 breast cancer cases diagnosed in the 1,702 women, the annual breast cancer incidence rate is 914/100,000 per year. The risk of breast cancer in the cohort was 4.9 times greater than the expected risk based on Polish national statistics (age‐adjusted) (Table 3).

**Table 2:** Incident cancers detected in the cohort

Cancer site	*n*	Mean arsenic level, μg/L (range)
Breast	68	1.54 (0.41–13.4)
Ovarian	6	8.78 (0.69–48.4)
Colon	5	1.02 (0.61–1.37)
Lymphoma	5	1.19 (0.75–1.89)
Uterus	5	1.08 (0.73–2.01)
Bladder	4	1.13 (0.81–1.82)
Thyroid	4	1.30 (0.89–1.58)
Cervix	2	0.81 (0.66–0.97)
Kidney	2	1.22 (1.19–1.24)
Leukemia	2	0.82 (0.65–0.99)
Melanoma	2	6.44 (1.07–11.8)
Myeloma	2	1.04 (0.95–1.12)
Endometrial	1	0.85
Lung	1	0.80
Meningioma	1	1.10
None	1,592	1.07 (0.04–20.1)

**Table 3:** Comparison of observed and expected numbers of breast cancers in the cohort

Age group	40–44	45–49	50–54	55–59	60–64	65–69	70–74	40–74
Person‐years	747.3	1,078.8	937.9	1,333.2	1,730.4	1,044.9	308.7	7,181.2
Background rate	70.2	116.9	152.5	163.9	213.7	258.9	178.6	
Expected cancers	0.52	1.26	1.43	2.19	3.70	2.71	0.55	12.4
Observed cancers	7	14	5	7	15	10	2	60
Observed cancer per 100,000	936.7	1,297.7	533.1	525.1	866.8	957.0	647.8	835.4
SIR	13.3	11.1	3.5	3.2	4.1	3.7	3.6	4.9

*Source*: Polish Cancer Registry.

Abbreviation: SIR, standardized incidence rate.

The 1,702 women were divided into four categories (quartiles) of equal size, based on their total blood arsenic level. The univariate and multivariate HRs of developing breast cancer with increasing quartile of arsenic levels are presented in Table 4. In the crude analysis, increasing arsenic levels were associated with a significantly increased risk of developing breast cancer (*p*‐trend <0.0001). Findings were similar in the analysis adjusted for age, smoking status (ever/never), number of first‐degree relatives with breast cancer (2 or more, 1 *vs*. 0), oophorectomy (yes/no) and hormone replacement therapy use (yes/no). In the adjusted analysis, women in the highest quartile of arsenic had a highly significant 13‐fold increased risk of developing breast cancer compared to women in the lowest quartile (HR = 13.2; 95% CI 4.02–43.0).

**Table 4:** Hazard ratio for breast cancer by blood level of arsenic (quartiles)

Arsenic level, μg/L	Total	Breast cancers	Univariate HR (95%CI)	*p*	Multivariate HR (95%CI)	*p*
<0.62	426	3	1.00 (ref)		1.00 (ref)	
0.62–0.82	425	15	5.21 (1.51–17.99)	0.01	5.11 (1.48–17.67)	0.01
0.82–1.19	426	17	6.13 (1.80–20.93)	0.004	6.51 (1.91–22.24)	0.003
>1.19	425	33	11.93 (3.66–38.91)	<0.0001	13.15 (4.02–43.03)	<0.0001

Multivariate hazard ratios are adjusted for age (>50 *vs*. ≤50), smoking status (ever/never), number of first degree relatives with breast cancer (2 and more, 1, *vs*. 0), oophorectomy (yes/no), and hormone replacement therapy use (yes/no). *p*‐Value for trend <0.0001.

**Figure 1:**
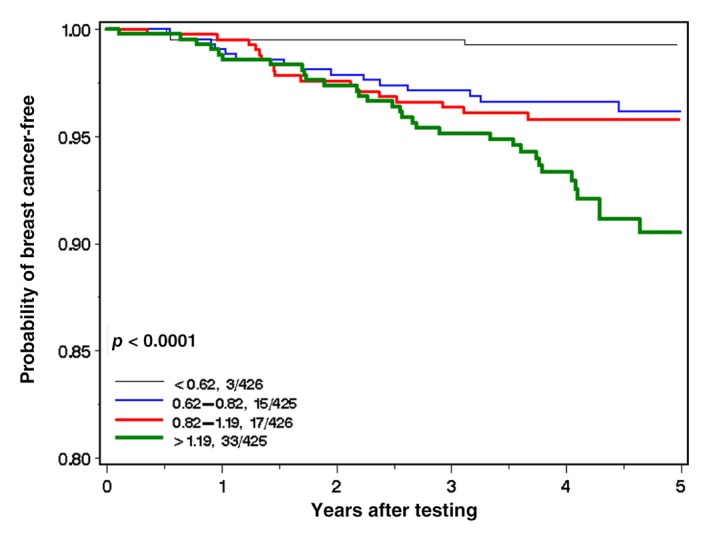
Probability of breast cancer‐free by arsenic level (quartiles). [Color figure can be viewed at http://wileyonlinelibrary.com]

The annual risk of breast cancer varied widely according to the blood arsenic level. For those in the first (lowest) quartile of blood arsenic levels, the annual risk was 152 per 100,000 per years; for those in the second quartile, the risk was 798 per 100,000 per year, for those in the third quartile the risk was 941 per 100,000 per year and for those in the highest quartile the risk was 1,855 per 100,000 per year. These incidence rates are depicted graphically in Figure 1. After 5 years of follow‐up, the cumulative incidence was 0.7% for quartile 1, 3.8% for quartile 2, 4.2% for quartile 3 and 9.5% for quartile 4 (Fig. 1).

**Figure 2:**
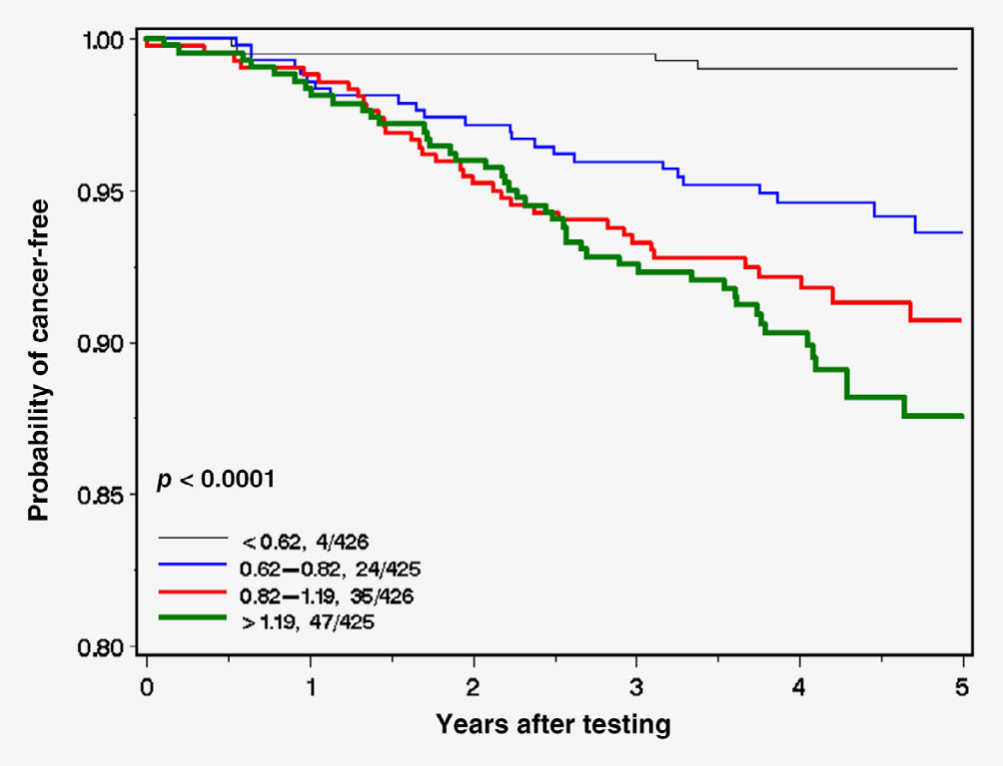
Probability of cancer‐free (any cancer) by blood arsenic level (quartiles). [Color figure can be viewed at http://wileyonlinelibrary.com]

In a secondary analysis, we considered all 110 cancers as the endpoint (Table 5). The results were similar. Women in the highest quartile of blood arsenic had a 13‐fold increased risk of developing any cancer compared to the lowest quartile (HR = 13.3; 95% CI 4.78–37.0). There was a significant difference in the cumulative incidence of any cancer with increasing quartile of blood arsenic (Fig. 2; *p*‐log rank test <0.0001).

**Table 5:** Hazard ratio for any cancer by blood level of arsenic (quartiles)

Arsenic level, μg/L	Total	Any cancer	Univariate HR (95%CI)	*p*	Multivariate HR (95%CI)	*p*
<0.62	426	4	1.00 (ref)		1.00 (ref)	
0.62–0.82	425	24	6.23 (2.16–17.95)	0.0007	5.83 (2.02–16.86)	0.001
0.82–1.19	426	35	9.48 (3.37–26.65)	<0.0001	9.77 (3.47–27.51)	<0.0001
>1.19	425	47	12.72 (4.58–35.30)	<0.0001	13.31 (4.78–37.02)	<0.0001

Multivariate hazard ratios are adjusted for age (>50 *vs*. ≤50), smoking status (ever/never), number of first degree relatives with breast cancer (2 and more, 1, *vs*. 0), oophorectomy (yes/no), and hormone replacement therapy use (yes/no). *p*‐Value for trend <0.0001.

## Discussion

In this prospective cohort study of Polish women at an elevated risk of breast cancer because of their family history, we found a strong and statistically significant association between baseline blood arsenic levels and the subsequent risk of breast cancer and with all cancers combined. There were only three breast cancers diagnosed among 426 women in the lowest quartile of arsenic (mean arsenic level 0.48 μg/L) compared to 33 cases diagnosed among the 425 women in the highest quartile (mean level 2.33 μg/L). This represents a HR of 13.2 and this association was highly significant (*p* < 0.0001). Similarly, in the analysis of all cancers combined, the HR comparing women in the highest *vs*. lowest quartile of blood arsenic was 13.3 (*p* < 0.0001). These findings suggest that in Poland blood arsenic status (even at low concentrations) is a strong predictor of breast cancer. This is a single study and needs to be repeated in Poland and elsewhere.

Given the strength of the associations reported here and the importance of the potential implications, it is important that we explore the possible reasons underlying these observations. We have considered the possibility that these are chance findings, but given the large effect sizes and associated *p*‐values, this possibility is unlikely. Furthermore, these findings remained relatively consistent in the multivariate model and we are not aware of other variables within the database (or variables not recorded) that may impact upon both arsenic levels and breast cancer risk. Importantly, we also observed a similar relationship between arsenic levels and all other cancers combined. With regard to multiple comparisons, we also evaluated the relationship between three other micronutrients (zinc, cadmium, selenium) and risk, but found no other significant associations (data not shown).

Arsenic compounds, which occur both naturally and as a result of human activity, can be divided into three types: organic, inorganic and arsine gas.^5^ All types of arsenic can contaminate ground and surface water that are commonly used to complete daily activities, such as drinking and cooking.^27^ The relative toxicity and/or carcinogenicity of each arsenic species has been evaluated; organic arsenic species and arsine gas are classified as potential carcinogens, and inorganic arsenic has been classified as a Group I carcinogen.^5^ There is growing evidence that long‐term, low‐level arsenic exposure might be harmful and carcinogenic.^28^, ^29^, ^30^ Potential mechanisms mediating the carcinogenic effects of arsenic include its impact on cellular differentiation and cellular proliferation as well as inducing chromosomal aberrations and sister chromatid exchange.^31^

Most of the epidemiologic evidence surrounding arsenic exposure has reported in terms of an increased risk of cancers of the lung, skin and bladder.^31^, ^32^ These associations are predominantly from studies that have evaluated the impact of contaminated drinking water.

In humans, primary arsenic exposure is primarily from ingestion of contaminated food or water, with less from inhalation and consumption of foods with low quantities (i.e., seafood, meats and cereal).^5^ Arsenic exposure through inhalation of arsine gas is an uncommon source of exposure for most populations.^33^ There is less of a concern of arsenic contamination of drinking water in Poland, thus, the main source of arsenic in our study population is likely *via* food consumption given that arsenic levels in water have been standardized to 10 μg/L for public drinking, in accordance with the World Health Organization standards.^34^ Thus, the main source of arsenic in our study population is likely *via* food consumption or previous consumption of contaminated water.^27^ In particular, seafood contains a variety of environmental contaminants, including mercury, arsenic and lead.^35^ Seafood and fish compounds peak interests due to their high total levels of arsenic; demersal and pelagic fish contain a high level of arsenobetaine a species of organic arsenic which is not known to be toxic as well as low levels of inorganic arsenic.^36^, ^37^

The mechanisms by which low arsenic levels increase the risk of breast cancer are unknown. In females, arsenic may influence development of cancers by disrupting the function of estrogen receptors and suppressing the signaling pathway of estrogen.^38^, ^39^ Arsenic is also a potential metallo‐estrogen.^40^ For example, the effects of estradiol are copied by sodium arsenite and this process leads to the proliferation of cells in an estrogen‐responsive breast cancer cell line.^41^ It is also possible that the observed association is the result of an unknown confounder that is another nutrient that is found in the same source as is arsenic and consumption of arsenic and the unknown carcinogen is highly correlated. Finally, we cannot preclude that blood levels of arsenic are correlated with some other physiologic process whereby accumulation of arsenic in the blood and breast cancer are two manifestations of the same process.

An association has been reported between polymorphisms in the arsenic methyltransferase gene (AS3MT) and arsenic‐related cancer risk.^42^ Multiple studies have demonstrated that incomplete arsenic metabolism, with higher fractions of inorganic arsenic and methyl‐arsenic acid and lower fractions of dimethyl‐arsenic acid, is a marker for increased susceptibility to arsenic‐related cancers.^5^, ^43^, ^44^ The methylation of arsenic is thought to be one of the primary aspects of arsenic carcinogenicity. Multiple studies have concluded that incomplete inorganic arsenic methylation causes the accumulation of toxic arsenic intermediate species in the blood and tissues.^45^, ^46^ This two‐step methylation process, when performed to completion, takes inorganic arsenic to monomethylarsonic acid first and then to dimethylarsinic acid, a substance that is easy to excrete and is relatively nontoxic.^45^ Mechanisms and theories that are currently accepted believe that incomplete methylation of inorganic arsenic to dimethylarsinic acid leads to the bioaccumulation of toxic arsenic species (such as inorganic arsenic and monomethylarsonic acid) in the body.^44^

There are several limitations associated with our study. We only had one blood sample available for arsenic quantification and were not able to assess reproducibility over time. Although one measure of arsenic may not reflect long‐term exposure, Smith *et al*. recently demonstrated significantly elevated rates of cancer mortality due to lung, bladder and kidney cancer up to 40 years after exposure to elevated arsenic levels in water suggestive of a long latency period between arsenic exposure and cancer development.^32^ On average, 4.5 years elapsed between the measurement of arsenic and the diagnosis of breast cancer (range 0.5–7.2 years); however, the blood specimens were stored in batches and cases and controls were stored for equal amounts of time and were all assayed on the same date. Furthermore, the technicians were blinded as to the clinical status of the patient. Additionally, we studied total blood arsenic as a measure of recent arsenic exposure. Future analysis should look at specific concentrations of arsenic species (inorganic [monomethylarsonic acid/dimethylarsinic acid/pentavalent arsenic ion] *vs*. organic) within total blood measurements. This analysis is crucial for further studies given the known carcinogenicity of the inorganic arsenic species. Moreover, it is known that arsenic has a short half‐life and is cleared from the blood within 3 to 6 hrs and thus our measurements represent a recent arsenic exposure.^47^ However, arsenic levels found in keratin rich tissues such as toenail or hair levels may better reflect long‐term exposure.^48^ Despite this, we chose total blood levels as a measure of internal dose of arsenic, so that it may better reflect the actual tissue burden compared to urinary, toenail or hair arsenic.^49–51^ Importantly, we have not explored the various factors whereby arsenic is absorbed, stored and eliminated and it is possible that one of these metabolic processes has a relationship to breast cancer risk as well. It may be that the findings are not generalizable to other populations because of other factors including family history, ethnicity, geographic variation or different sources of arsenic (i.e., food or environmental conditions).

In summary, chronic low‐level exposure to arsenic compounds may lead to more than 10‐fold increased breast and all other cancer risk in Polish females. Unexpectedly, the blood arsenic level may be particularly strong marker of low/high cancer risk in women. For validation of above findings, further investigations on additional groups of females from Poland and other countries are needed.

## Data Availability

The data that support the findings of our study are available from the corresponding author upon reasonable request.
